# Latest updates on COVID-19 from the European Centre for Disease Prevention and Control

**DOI:** 10.2807/1560-7917.ES.2020.25.6.2002131

**Published:** 2020-02-13

**Authors:** Eurosurveillance editorial team

**Affiliations:** 1 European Centre for Disease Prevention and Control (ECDC), Stockholm, Sweden

The European Centre for Disease Prevention and Control (ECDC) provides regularly updated information relevant to Europe through a dedicated webpage. Below we highlight the latest additions.

## Guidelines for the use of non-pharmaceutical measures to delay and mitigate the impact of 2019-nCoV

This document provides guidance on the application of non-pharmaceutical countermeasures to minimise the spread of the 2019 novel coronavirus (2019-nCoV) in the population. Some of the measures proposed refer specifically to certain phases of the epidemic (containment or mitigation phases), and can be adapted depending on the assessed severity/impact of the infection. Other measures are valid for all phases of an epidemic. The guidance is based on the current knowledge of the 2019-nCoV and evidence available on other viral respiratory pathogens, mainly the Severe Acute Respiratory Syndrome coronavirus (SARS-CoV), the Middle East Respiratory Syndrome-related coronavirus (MERS-CoV) and seasonal or pandemic influenza viruses. ECDC will update this guidance as and when new relevant information becomes available or as required by the epidemiological situation.

## Interim guidance for environmental cleaning in non-healthcare facilities exposed to 2019-nCoV

The aim of this document is to support public health preparedness planning with regard to personal protective equipment (PPE) needs in healthcare settings where patients suspected or confirmed to have been infected with the novel coronavirus 2019-nCoV are being treated.

## Infographic: Novel coronavirus

This infographic provides basic information about the virus, symptoms, prevention and transmission.

**Figure:**
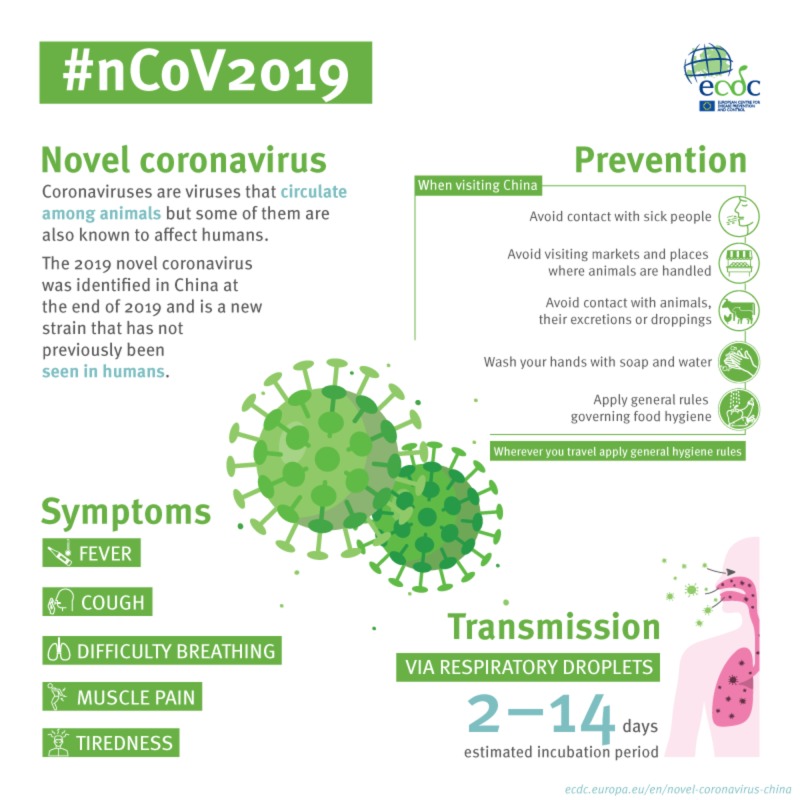
Infographic

We would also like to remind the readers of *Eurosurveillance* about our dedicated ‘Coronavirus disease 2019 (COVID-19) collection’ that we update regularly with new articles. The most recent addition was ‘First cases of coronavirus disease 2019 (COVID-19) in France: surveillance, investigations and control measures, January 2020’ while it already contained several articles about, for example, the development of a diagnostic methodology based on RT-PCR without the need for virus material, the severity among hospitalised cases, early-stage estimates of the importation risk to Europe, and estimated patterns of early human-to-human transmission.

